# Acute-On-Chronic Liver Failure: Current Interventional Treatment Options and Future Challenges

**DOI:** 10.3390/jpm13071052

**Published:** 2023-06-26

**Authors:** Markus Kimmann, Jonel Trebicka

**Affiliations:** 1 Department of Internal Medicine B, University of Münster, 48149 Münster, Germany; 2 European Foundation for the Study of Chronic Liver Failure, EFCLIF, 08021 Barcelona, Spain

**Keywords:** hepatology, liver cirrhosis, liver transplantation, decompensated cirrhosis, acute-on-chronic liver failure

## Abstract

Acute-on-chronic liver failure (ACLF) is a frequent complication in patients with liver cirrhosis that has high short-term mortality. It is characterized by acute decompensation (AD) of liver cirrhosis, intra- and extrahepatic organ failure, and severe systemic inflammation (SI). In the recent past, several studies have investigated the management of this group of patients. Identification and treatment of precipitants of decompensation and ACLF play an important role, and management of the respective intra- and extrahepatic organ failures is essential. However, no specific treatment for ACLF has been established to date, and the only curative treatment option currently available for these patients is liver transplantation (LT). It has been shown that ACLF patients are at severe risk of waitlist mortality, and post-LT survival rates are high, making ACLF patients suitable candidates for LT. However, only a limited number of patients are eligible for LT due to related contraindications such as uncontrolled infections. In this case, bridging strategies (e.g., extracorporeal organ support systems) are required. Further therapeutic approaches have recently been developed and evaluated. Thus, this review focuses on current management and potential future treatment options.

## 1. Introduction

Liver cirrhosis is associated with high morbidity and mortality, and represents a considerable public healthcare burden worldwide [1,2,3]. A recent study by Gu et al. investigated the epidemiology of cirrhosis in Germany. It was shown that cirrhosis was diagnosed in 0.94% of all patients admitted to hospital. Remarkably, 54.8% of these patients were diagnosed with cirrhosis or cirrhosis-related complications as a comorbidity, while the primary reasons for hospital admission were other diagnoses. Alcoholic liver cirrhosis accounted for 52% of admissions with cirrhosis [1]. Patients with liver cirrhosis are at risk of acute decompensation (AD), which is defined by the occurrence of cirrhosis-related complications and hospitalization, and it is associated with increased mortality [4]. The prevalence of different complications of cirrhosis has changed over time, with a decrease in bleeding complications and an increase in the prevalence of portal vein thrombosis (PVT), infections, hepatic encephalopathy (HE), ascites, hepatorenal syndrome, and hepatocellular carcinoma [1]. Recent studies have investigated the clinical course of AD and identified distinct clinical phenotypes ranging from stable decompensated cirrhosis (SDC) to unstable decompensated cirrhosis (UDC) to pre-ACLF patients [5,6]. The latter develop acute-on-chronic liver failure (ACLF), which is especially driven by severe systemic inflammation (SI) [7,8] within 90 days. According to the EASL definition, ACLF is defined by intra- and extrahepatic organ failure in patients with acutely decompensated liver cirrhosis, and is associated with a 28-day mortality rate in about 30% of cases [9]. Recent studies have demonstrated a high prevalence of ACLF in patients hospitalized due to AD worldwide [10]. Different precipitants for AD have been identified, i.e., bacterial infections, severe alcoholic hepatitis, bleeding with shock, and drug-induced toxic encephalopathy [11]. Currently, management of these patients consists of identification and treatment of the precipitant as well as the respective intra- and extrahepatic organ failures. However, no specific therapy exists for this group of patients, and to date the only curative treatment is liver transplantation (LT). On the one hand, there is a scarcity of donor organs because of strong competition for patients on the waiting list, while on the other hand, in terms of eligibility, ACLF patients may present with contraindications for LT, i.e., uncontrolled bacterial infection. Extracorporeal liver support systems (ECLS) such as albumin dialysis have been and are currently being evaluated, and new approaches and experimental therapeutic strategies are being tested.

## 2. Precipitants and Definition of AD and ACLF

AD in patients with liver cirrhosis is defined by the occurrence of cirrhosis-related complications such as acute gastrointestinal bleeding, development of ascites, HE, and bacterial infection, which lead to hospital admission [6]. Development of AD based on precipitants, i.e., bacterial infection, alcohol-induced hepatitis, gastrointestinal bleeding, and drug induced toxic encephalopathy, marks a crucial time point in the clinical course of patients with liver cirrhosis [12]. Interestingly, the PREDICT study found that more than 96% of patients with precipitants showed proven bacterial infection and/or severe alcoholic hepatitis. However, no precipitant was identified in 39% of patients with AD who developed ACLF. Bacterial translocation might play a role in this group of patients. Furthermore, it was shown that the number of events, not the type of event, seemed to determine prognosis in these patients [11]. A recent meta-analysis showed that bacterial infections were the most common precipitants worldwide [10]. However, there were regional differences, with alcohol-induced hepatitis being the most common precipitant in East Asia and North America. In particular, the first episode of AD marks a shift towards end-stage cirrhosis leading to a significant reduction in terms of median survival time. Around 30% of patients with AD develop intra- or extrahepatic organ failures and suffer from severe systemic inflammatory response [13]. However, due to divergent definitions of ACLF, cautious interpretation is needed when comparing international studies and data. These definitions and characteristics are summarized in Table 1.

The Chronic Liver Failure Acute-on-Chronic Liver Failure in Cirrhosis (CANONIC) study was able to determine major risk factors for mortality in patients presenting with AD, and defined ACLF as a distinct syndrome in patients with decompensated cirrhosis [9]. Based on these findings, the Chronic Liver Failure–Sequential Organ Failure Assessment (CLIF-SOFA) score and the Chronic Liver Failure Consortium Organ Failure (CLIF-C OF) score were developed to assess ACLF in patients. According to the European Association for the Study of the Liver Chronic Liver Failure (EASL-CLIF) Consortium definition, ACLF is present in patients with acutely decompensated liver cirrhosis with single kidney failure (serum creatinine ≥ 2 mg/dL) or single organ failure combined with kidney dysfunction (serum creatinine range 1.5–1.9 mg/dL) and/or mild-to-moderate HE or presence of two or more organ failures [14].

The North American Consortium for the Study of End-Stage Liver Disease (NACSELD) defines ACLF as a condition in patients with acutely decompensated cirrhosis with or without prior episode(s) of decompensation and two or more organ system failures (maximum of four organ failures) that is associated with increased mortality within three months in the absence of treatment of the underlying liver disease, liver support, or LT. The organ failures that are taken into account include kidney (need for renal replacement therapy), brain (HE grade III or IV according to the West Haven Criteria), circulation (shock, mean arterial pressure < 60 mmHg), and respiration (need for mechanical ventilation) [15].

**Table 1:** Different definitions and characteristics of Acute-on-chronic liver failure.

	Asian Pacific Association for the Study of the Liver (APASL) ACLF Research Consortium (AARC) [16]	European Association for the Study of the Liver-Chronic Liver Failure (EASL-CLIF) Consortium [9]	North American Consortium for the Study of End-Stage Liver Disease (NASCELD) [15]
Patients considered in the definition	Acute liver deterioration in patients with diagnosed or undiagnosed chronic liver disease (including liver cirrhosis) Both compensated cirrhosis and non-cirrhotic chronic liver disease (chronic hepatitis with fibrosis, or fibrosis due to other reasons, non-alcoholic fatty liver disease, related chronic hepatic injury) qualify as chronic liver disease	Patients with an acute decompensation of liver cirrhosis with or without prior episode(s) of decompensation	Patients with an acute decompensation of liver cirrhosis with or without prior episode(s) of decompensation
Precipitating disorders	Extrahepatic (bacterial infection), intrahepatic (HBV reactivation), or both	Extrahepatic (infection, gastrointestinal bleeding), intrahepatic (alcoholic hepatitis), or both	Extrahepatic (infection)
Definition	ACLF definition is based on the presence of liver dysfunction. Extrahepatic organ failures may develop but are not included in the definition	ACLF definition is based on the existence of the failure of 1 or more of the 6 major organ considered in the CLIF-C Organ Failure scale (organ systems considered: Liver, kidney, brain, coagulation, circulation, respiration)	ACLF definition is based on the existence of 2 organ system failures or more (maximum 4) according to the NASCELD definition (organ systems considered: kidney, brain, circulation and respiration)
Stratification of ACLF	Jaundice (total bilirubin levels of 5 mg/dL or more) and coagulopathy (INR of 1.5 or more, or prothrombin activity of less than 40%) as a result of an acute hepatic insult which is complicated within 4 weeks by ascites, encephalopathy, or both. The severity of ACLF is assessed using the AARC score	ACLF is divided into 3 grades with increasing severity and mortality. ACLF grade 1 includes: - patients with single kidney failure - patients with single liver, coagulation, circulatory or lung failure that is associated with creatinine levels ranging from 1.5 mg/dL to 1.9 mg/dL or hepatic encephalopathy grade 1 or grade 2, or both - patients with single brain failure with creatinine levels ranging from 1.5 mg/dL to 1.9 mg/dL ACLF grade 2 includes: - patients with 2 organ failures ACLF grade 3 includes: - patients with 3 organ failures or more had ACLF grade 3	The stratification of patients is based according to the number of organ failures 2, 3, or all 4 organ failures, respectively
Approximate short-term mortality according to the stratification of ACLF	By 28 days: Grade 1: 13% Grade 2: 45% Grade 3: 86%	By 28 days: Grade 1: 23% Grade 2: 31% Grade 3: 74%	By 30 days: 2 organ failures: 18 to 43% 3 organ failures: 45 to 68% 4 organ failures: 77%

The Asian Pacific Association for the Study of the Liver (APASL) defines ACLF as an acute hepatic insult with jaundice (defined as serum bilirubin ≥ 5mg/dL) and coagulopathy (defined by international normalized ratio (INR) ≥ 1.5) complicated within four weeks by clinically manifestation of ascites and/or HE in patients with or without diagnosis of chronic liver disease and cirrhosis and a high 28-day mortality; organ failures are not included in the definition. The grade of ACLF is assessed using the AARC scoring system, which includes bilirubin, HE grade, INR, lactate, and creatinine [16].

## 3. Clinical Courses and Pathophysiology of AD

The CANONIC and PREDICT studies provided data on the role of SI as a crucial determinant in the development of cirrhosis-related complications and organ failure in addition to the well-known role of portal hypertension (PHT) and its complications, supporting the systemic inflammation hypothesis [8]. Furthermore, it has been shown that SI is associated with disease severity as well as with patient survival rates.

The PREDICT study was able to show that AD is characterized by different clinical phenotypes. These phenotypes depict heterogenous clinical conditions with distinct pathophysiology and a different prognosis. Therefore, the authors of the PREDICT study suggested a novel classification into three patient groups to better identify and differentiate these distinct courses of AD.

Most patients admitted with AD belong to the group of patients with SDC, who show low SI, are more likely to be recompensated quickly, show fewer cirrhosis-associated complications, and have the lowest 1-year mortality risk of the three groups.

The second clinical course of AD suggested by the PREDICT study is UDC. These patients mainly suffer from PHT-driven complications, present with a high prevalence of bacterial infections, i.e., spontaneous bacterial peritonitis, and have a higher risk of further decompensation and significantly decreased survival rates. In terms of pathophysiology, severe PHT seems much more relevant than in the other courses of AD, while SI is present in these patients as well.

Lastly, there are patients with AD who are characterized by development of ACLF within 90 days, constituting the group of pre-ACLF patients. These patients show severe progression of SI, possibly a key factor in development of intra- and extrahepatic organ failures, and show the highest short-term mortality among the three groups [6].

**Figure 1:**
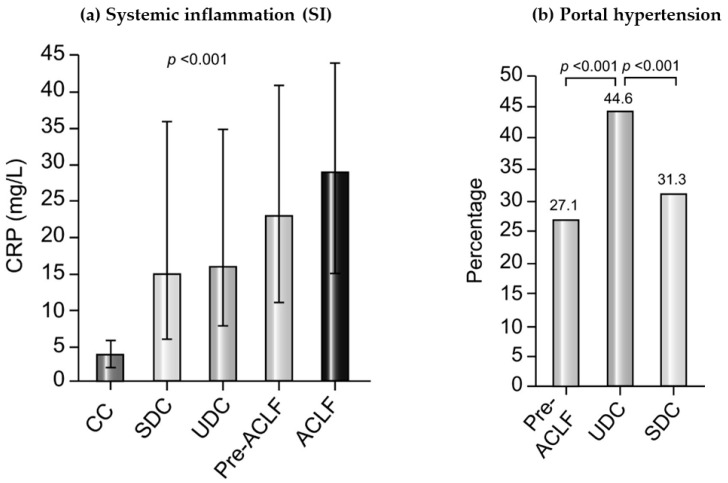
The role of Systemic inflammation (SI) and Portal Hypertension in different stages of cirrhosis according to the results of the PREDICT study. (**a**) Plasma levels of CRP as a marker of SI in patients with compensated cirrhosis (CC, no prior history of AD), SDC, UDC, pre-ACLF, and ACLF. *p* values were obtained using Kruskal–Wallis test. (**b**) Percentage of patients presenting at least one surrogate of severe portal hypertension during the 6-month observational period of the PREDICT study in the Pre-ACLF, UDC, and SDC groups. *p* values were obtained using chi-square test. ACLF, acute-on-chronic liver failure; AD, acute decompensation; CC, compensated cirrhosis; CRP, C-reactive protein; SDC, stable decompensated cirrhosis; UDC, unstable decompensated cirrhosis. Figure modified from Trebicka et al., 2020 [6].

Overall, both PHT and SI are important in the pathophysiology of AD, with different characteristics depending on the clinical course of AD. The results of the PREDICT study suggest that the relevance of SI increases over the clinical course of AD and is most prominent in pre-ACLF and ACLF patients, while PHT is especially relevant in UDC patients [6] (Figure 1).

## 4. Management of AD and ACLF

### 4.1. Prediction of Decompensation

The early detection of patients at risk of decompensation is essential to allow early treatment and optimize their prognosis. The ANTICIPATE study highlighted the diagnostic value of liver stiffness measurements (LSM) in combination with platelet count for identifying clinically significant portal hypertension [17]. The Baveno VII consensus further highlighted the diagnostic value of LSM and stated the “rule of five” for LSM by the transient elastography (10–15–20–25 kPa) approach to denote higher relative risk of decompensation and liver-related death [18]. Additionally, a recent international multicenter cohort study provided an efficient and simple algorithm (M10LS20 algorithm) for risk stratification of patients with chronic liver disease that was externally validated. This algorithm consists of the Model of End Stage Liver Disease (MELD) score and LSM by shear wave elastography. A combined cutoff of a MELD score ≥ 10 and a liver stiffness of ≥20 kPa was able to identify those patients with a poor prognosis who have high mortality and risk of development or worsening of decompensation [19].

### 4.2. Prevention of AD and ACLF

Several therapeutic options have been evaluated for prevention of decompensation and for the management of patients with liver cirrhosis.

The role of non-selective beta blockers (NSBB) has been the topic of research in the recent past. In the PREDESCI study on patients with compensated cirrhosis and clinically significant portal hypertension the use of NSBB was associated with increased decompensation-free survival, mainly by reducing the incidence of ascites [20].

Aspirin should not be discouraged in patients with an approved indication, as it may reduce the risk of hepatocellular carcinoma, death, and liver-related complications [18,21]. The same applies for anticoagulation in patients with an approved indication, as it may improve the prognosis of these patients [18,22].

The use of statins in patients with liver cirrhosis and an indication for statins has been shown to improve survival, and may even decrease portal pressure [23]. Furthermore, Mahmud et al. were able to demonstrate that statin use and the duration of therapy significantly reduced the risk of ACLF [24]. However, statins should be used at a lower dose (simvastatin at max. 20 mg/d) in patients with Child–Pugh B and C cirrhosis due to a higher rate of adverse events, and patients should be followed for muscle and liver toxicity [25]. It remains unclear whether patients with decompensated liver cirrhosis without indication for statins benefit from this medication, and more data is necessary. Currently, we are awaiting the results of a phase III multicenter double-blind placebo-controlled randomized clinical trial (NCT03780673) investigating the use of simvastatin plus rifaximin in patients with decompensated cirrhosis to prevent ACLF.

Antibiotic prophylaxis is a keystone in preventing bacterial infections, and especially in preventing spontaneous bacterial peritonitis (SBP), which are both common precipitants of AD and ACLF. Thus, primary antibiotic prophylaxis is recommended in patients with gastrointestinal bleeding and Child–Pugh C cirrhosis with low protein ascites, as they are at high risk of SBP development. In patients with a history of previous SBP, secondary antibiotic prophylaxis is indicated [18,26].

However, while rifaximin is not indicated for primary or secondary prophylaxis of SBP, it is indicated for secondary prophylaxis of HE, and should be considered in patients undergoing elective transjugular intrahepatic portosystemic shunt (TIPS) implantation who have a history of overt HE [18].

The role of albumin administration, which has been discussed as a disease modifying agent, is a topic of current research. In this context, short-term and long-term administration of albumin must be distinguished.

In patients with spontaneous bacterial peritonitis (SBP) and specific risk factors (bilirubin > 4 mg/dL or creatinine > 1 mg/dL), albumin infusion has been shown to decrease the risk of acute kidney injury, and has been associated with improved prognosis [27].

According to the Baveno VII consortium, there is an indication for short-term albumin administration in patients with SBP, acute kidney injury (AKI), large-volume paracentesis (>5 L), and, in combination with terlipressin, in the treatment of hepatorenal syndrome (HRS) [18]. Furthermore, a recent study by Arora et al. demonstrated that albumin infusion decreases the incidence of paracentesis-induced circulatory dysfunction and mortality in ACLF patients receiving paracentesis < 5 L [28].

The role of long-term albumin administration is more controversial. The multicenter open-label ATTIRE study by China et al. found that targeted albumin infusion in patients hospitalized with liver cirrhosis and serum albumin levels < 30 g/L did not decrease the risk of onset of infection, acute kidney injury, or death [29].

However, the multicenter open-label ANSWER study investigated the effect of long-term albumin administration (40 g twice weekly for two weeks and then 40 g weekly for up to 18 months) in patients with decompensated cirrhosis and uncomplicated ascites, and showed increased overall survival in the group of patients with long-term albumin administration. Currently, no clear recommendation regarding long-term albumin administration can be provided due to insufficient data.

In this regard, the Prevention of Mortality with Long-Term Administration of Human Albumin in Subjects With Decompensated Cirrhosis and Ascites (PRECIOSA) trial (NCT03451292), which is currently recruiting, will hopefully improve the evidence base regarding long-term administration of albumin. Furthermore, a study on personalized long-term albumin treatment in patients with decompensated cirrhosis and ascites (Alb-trial) (NCT05056220) guided by the MICROB-PREDICT biomarker is expected to provide more evidence.

### 4.3. Treatment of the Precipitants of AD and ACLF

As a result of the lack of currently available specific treatment options for AD and ACLF, management consists of identifying, preventing, and treating precipitants of ACLF as well as management of the respective intra- and extrahepatic organ failures [26,30].

If a precipitant is ascertainable, early detection and adequate treatment are both crucial.

The PREDICT study demonstrated that the most frequent precipitants in Europe are bacterial infections and severe alcoholic hepatitis, and that the number of precipitants is relevant, the latter being significantly associated with surrogates for SI and increased 90-day mortality [11].

Liver cirrhosis is associated with dysfunctions of various components of the immune system, which together have been described as cirrhosis-associated immune dysfunction [31]. Patients with liver cirrhosis have an increased risk of developing bacterial infections, which are the most common precipitants of ACLF in Europe [11]. If a bacterial infection is confirmed, it is essential to begin timely and adequate antibiotic treatment, which has been shown to decrease the incidence of ACLF development in patients with AD and to decrease mortality in patients with ACLF [32,33]. The most common bacterial infection in this context is SBP [34]. Due to the increasing prevalence of multidrug-resistant organisms (MDRO), the high prevalence of these organisms in patients with ACLF, and their lower infection resolution rates, antibiotic treatment of these patients is challenging [32]. Interestingly, antibiotic prophylaxis of SBP with norfloxacin was not associated with an increased incidence of MDRO [35]. However, it is crucial to initially start a broad empirical antibiotic treatment that takes into account the local resistance spectrum as well as the recent medical history of the patient (e.g., recent interventions such as ascites drainage) and to subsequently adjust the therapy according to microbiological results [35,36].

A recent study by Fernandez et al. investigated the effect of albumin administration in patients with advanced cirrhosis and non-SBP infections. While there was no difference in terms of in-hospital mortality, in the group of patients who received albumin there was a higher rate of ACLF resolution and a lower proportion of nosocomial infections despite the fact that the group of patients who received albumin were more seriously ill at baseline [32]. Thus, albumin administration in this subgroup of patients might be beneficial.

Another common trigger for ACLF is severe alcoholic hepatitis, which is linked to massive inflammation. The STOPAH trial investigated therapeutic approaches (pentoxifylline and prednisolone) for these patients and found no significant survival benefit for either drug. However, there was a benefit found in the 28-day survival rate in the group of patients who received prednisolone [32], possibly due to the higher likelihood of infections being developed during the additional observation period. According to the relevant EASL guideline, prednisolone 40 mg/day should be administered in patients with a Glasgow Alcoholic Hepatitis score (GAHS) of ≥9 [37]. GAHS was developed to identify patients at risk of death in case of alcoholic hepatitis [38]. After seven days of prednisolone therapy, treatment response is assessed by the Lille model for alcoholic hepatitis, which is a risk stratification tool for patients with alcoholic hepatitis receiving steroids for seven days [39]. In patients with a Lille score < 0.45, prednisolone administration should be continued for a total of 28 days, while in patients with a Lille score > 0.45 prednisolone should be discontinued. Patients with severe alcoholic hepatitis who receive prednisolone therapy have significantly increased mortality in terms of infection. Therefore, if an infection is already present, prednisolone therapy should not be administered when severe alcoholic hepatitis is present [37]. Furthermore, a French multicenter study suggests that in severe alcoholic hepatitis the combination of N-acetylcysteine with prednisolone has a better 1-month survival and a lower incidence of renal failure and infection compared with prednisolone therapy alone. However, 6-month survival was not shown to be different [35].

It has to be taken into account that the number of organ failures at admission, which itself indicates the severity and grade of ACLF, is negatively correlated with response rates to prednisolone therapy [40]. In most allocation systems, patients with alcohol-related liver disease need to be abstinent for at least six months in order to be considered for LT. However, Mathurin et al. evaluated the option of LT in patients with a first episode of severe alcoholic hepatitis not responding to medical therapy who failed to be abstinent for at least six months, demonstrating that in selected patients early transplantation can improve survival [41]. Another recent multicenter non-randomized study confirmed the survival benefit related to early liver transplantation for severe alcoholic hepatitis, showing similar 2-year survival rates for the early and standard (six months of alcohol abstinence) transplantation groups. However, non-inferiority in terms of rate of alcohol relapse post-transplant between early liver transplantation and standard transplantation could not be concluded [42]. Overall, this topic remains controversial.

Another trigger of ACLF is upper gastrointestinal bleeding, which is a potentially acute life-threatening event [43]. The management of upper gastrointestinal bleeding, whether variceal or nonvariceal, first consists of monitoring the patient and providing hemodynamic and respiratory stabilization. Volume replacement with crystalloid fluids and, if necessary, catecholamine therapy and transfusion (restrictively from Hemoglobin < 7 g/dL) play a major role in the management of these patients [26,44]. Administration of an initial bolus of proton-pump inhibitor (PPI) seems reasonable, as up to 50% of upper gastrointestinal bleedings in patients with liver cirrhosis are not varicose. However, if purely variceal bleeding is confirmed there is no indication for long-term PPI administration. Because the size of postligation ulcers is reduced by PPI therapy, short-term administration should be considered [26]. In suspected variceal bleeding, intravenous therapy with vasoconstrictors of the splanchnic area (terlipressin, somatostatin, and octreotide) should be initiated immediately and even before endoscopy. In confirmed variceal bleeding this therapy should be continued for a total of five days to prevent recurrent bleeding. Terlipressin should be administered initially as a bolus and ideally continuously during the course of therapy [26,45]. Furthermore, immediate antibiotic therapy (e.g., third generation cephalosporines), usually for seven days, should be administered, as it improves bleeding control and survival and is associated with a reduced rate of recurrent bleeding [46]. Interestingly, bacterial infections are present in about 50% of patients with acute esophageal variceal bleeding, and are often precipitants of the bleeding [46]. Endoscopic diagnosis and therapy should be performed within the first 12 h after hemodynamic stabilization. However, up to 15% of patients develop recurrent bleeding [26]. Regarding indication for protective endotracheal intubation, there are no safe intubation criteria except for coma and suspected airway obstruction. The European Association of Gastrointestinal Endoscopy (ESGE) suggests endotracheal intubation before endoscopy in patients with active hematemesis, encephalopathy, or agitation [47].

In high-risk patients (Child–Pugh B and active bleeding on screening endoscopy or Child–Pugh C < 14 points), early TIPS (within the first 24–72 h) can be considered primarily, while in the case of recurrent bleeding it should be evaluated secondarily [18]. A recent multicenter international observational study identified ACLF at admission as an independent predictor of rebleeding and mortality in patients with acute variceal bleeding. Furthermore, preemptive TIPS placement was associated with improved survival (42-day and 1-year survival) in patients with ACLF and acute variceal bleeding, which indicates the important role of portal hypertension in these patients [48].

### 4.4. Organ Liver Support in ACLF

ACLF is associated with intra- and extrahepatic organ failures, and the supportive treatment of these respective organ failures plays an important part in the management of these patients. A frequent syndrome that occurs in decompensated cirrhosis is HRS-AKI. It is defined by the International Ascites Club as AKI in patients with cirrhosis, acute liver failure, or acute-on-chronic liver failure which does not show full or partial response after at least two days of diuretic withdrawal and volume expansion with albumin (1 g/kg of body weight per day to a maximum of 100 g/day) in the absence of shock, treatment with nephrotoxic drugs, and absence of parenchymal kidney disease [49]. Vasoconstrictors (e.g., terlipressin) and albumin are considered first line therapy for patients with HRS-AKI [26].

Renal replacement alone is useful in the short term for acute renal failure requiring dialysis; however, it has not been shown to be suitable as a medium- or long-term option in HRS [50]. In the context of liver failure and ACLF, both toxic hydrophilic substances which could be removed from the circulation by conventional dialysis and non-hydrophilic substances accumulate in the body [51]. Thus, ECLS systems have been developed that can eliminate albumin-bound substances. In the past, two large randomized controlled trials (HELIOS and RELIEF) which evaluated the influence of two different ECLS systems (MARS^®^ and Prometheus^®^) in ACLF patients were unable to show a significant benefit in overall survival in the overall cohort [45,46]. However, at least in the HELIOS trial, the subgroup of patients with a MELD score > 30 showed improved survival [52]. A recent meta-analysis of 25 randomized controlled trials of ELCS systems showed at least a moderate certainty regarding reduction of mortality (RR 0.84, 95%CI 0.74–0.96) [53]. Furthermore, another meta-analysis of individual patient data investigated the use of MARS^®^ and treatment intensity in ACLF, finding survival benefits in the group of patients who received high-intensity therapy (more than four MARS^®^ sessions), especially in the first ten days [54]. Another recent meta-analysis which included 16 randomized controlled trials concluded that of all support systems for ACLF patients, plasma exchange might be the best current treatment option [55]. Currently, the APACHE trial, a large randomized controlled trial, is being performed to provide more evidence on plasma exchange. A general recommendation for the use of any ECLS in ACLF patients cannot be provided due to the lack of sufficient evidence. However, in selected cases or in the context of clinical trials ECLS systems should be considered as a therapeutic option, especially as a bridging strategy to improve short-term survival in patients who are potentially eligible for LT [25]. The results of current and future trials will identify patients who could benefit from ECLS.

### 4.5. LT in AD and ACLF

Although prevention and timely adequate treatment of precipitants of ACLF is essential even when supportive treatment of the respective intra- and extrahepatic organ failures is performed, the only curative and potentially life-saving therapeutic option for patients with AD and ACLF remains LT.

Recent data have generated a consensus that patients with ACLF, especially grades 1 and 2, should be listed for LT and benefit from timely evaluation for LT. This concept is supported by the fact that even ACLF patients who recover from their ACLF episode are at high risk of future decompensation and ensuing ACLF development, and suffer from high mortality [48]. Recently, it has been shown by Sundaram et al. that ACLF patients, especially ACLF grade 3 patients, have high waitlist mortality. At the same time, 1-year survival rates in ACLF patients who received LT were not significantly different from patients without ACLF in this study [56]. However, there are uncertainties regarding timing and selection of patients for LT, especially in case of ACLF grade 3 patients. For example, in patients with ACLF grade 3 and PVT, mortality was significantly higher than in ACLF grade 3 patients without PVT, indicating that LT should be approached cautiously in this subgroup of patients [56]. Interestingly, a recent publication by Zhang et al. demonstrated that in ACLF grade 3 patients earlier transplantation improves survival even if the organ is suboptimal. In particular, in the first week the use of borderline organs is significantly more advantageous than waiting for the patient to achieve a lower ACLF grade or to regain organ function. This effect was particularly noticeable in patients older than 60 years or with 4–6 organ failures [57].

The PREDICT study was able to demonstrate that UDC and pre-ACLF patients have higher short-term mortality than patients with SDC. These patients are at high risk of progression to ACLF and development of organ failure, which is associated with potentially rapid clinical deterioration and even higher short-term mortality [6].

The current scoring tools used for liver transplant allocation, such as MELD [58], MELD-sodium (MELD-Na) [59], and Child–Pugh score [60], may not adequately reflect the high short-term mortality of ACLF patients. These models have been widely discussed in the recent past, and it is known that different clinical conditions (e.g., frailty, sarcopenia, recurrent HE) are not adequately reflected by the underlying scores. Regarding ACLF, the scores have no surrogate for SI, i.e., CRP, ferritin, or white blood cell count (which is the main pathophysiological driver of ACLF progression and has been found to be strongly correlated with mortality rate in these patients) [7,61,62]. Additionally, neither the MELD score nor the MELD-Na score includes markers for portal hypertension, which is the main driver in UDC and is a relevant factor in the development of complications such as acute variceal bleeding or development of ascites and SBP in these patients. Limitations of the current risk stratification allocation policies are further underlined by a recent study of Sundaram et al. which demonstrated that patients with ACLF grade 3 and MELD-Na < 25 have higher mortality than patients with MELD-Na > 35 without ACLF [56]. Hernaez et al. were able to demonstrate that the MELD-Na score distinctly underestimates the 90-day mortality of ACLF patients [63].

In an awareness of these limitations that are especially relevant for patient with ACLF, the CLIF-C-ACLF score was specifically more than adequate in rating the mortality risk in ACLF patients. This score consists of the number of organ failures, which are reflected by the CLIF-OF score, age and the white blood cell count, which is used as the parameter indicating the severity of SI [64]. Each of these parameters has been investigated as a predictor of mortality in ACLF patients. The predictive accuracy of the CLIF-C ACLF score, especially in terms of short-term mortality in ACLF patients, has been affirmed in the recent past, and has been shown to be superior to other prognostic models in ACLF patients [64,65]. Furthermore, a CLIF-C ACLF score of 64 points or above is regarded as the threshold of futility of care, and is used as a tool to identify patients for whom supportive care has to be critically discussed if LT is not a valid option [66]. A recent publication by Schulz et al. found that pulmonary impairment independently determined mortality in critically ill patients with ACLF. They proposed the CLIF-C ACLF-R score, which is a modified CLIF-C ACLF score that uses a calibration variable to adjust for the presence or absence of mechanical ventilation or pulmonary failure. After further external validation, this simple modification could be used in clinical practice to improve the stratification of these patients [67]. Another recent publication by Weiss et al. investigated the role of metabolites reflecting SI, mitochondrial dysfunction, and sympathetic system activation in predicting short term mortality in patients with ACLF, and invented the CLIF-C MET score. However, cost-effectiveness analysis and prospective validation of these markers and scores remains necessary [68].

Overall, these findings advocate for the need to discuss the necessity of improving and modifying the current allocation systems to better reflect waitlist mortality, and especially to adequately reflect the prognosis of ACLF patients.

## 5. Outlook for Possible Future Therapeutic Options for ACLF Management of AD and ACLF

To date, no drug has been approved as a specific treatment for ACLF. However, different therapeutic options are evaluated here.

Granulocyte-colony stimulating factor (G-CSF) was considered a novel therapy for patients with ACLF, and showed promising results in small single center studies. However, a multicenter randomized phase-II trial by Engelmann et al. revealed that it improved neither patient survival rates nor organ function and that it failed to reduce the rate of complications [69]. Yet, based on promising results in experimental mouse models (inhibition of inflammation and promoting hepatocyte regeneration) [70], the combination of G-CSF and TAK-242 (an inhibitor of Toll-like receptor-4) in ACLF patients is scheduled to be investigated in a randomized trial (EU-funded A-TANGO project).

Omega-3 fatty acids as a treatment option for ACLF were investigated in a small open-label randomized controlled trial of 90 patients. The patients were randomized into three groups (1, regular diet; 2, regular diet plus 50 mL/d of intralipid 20% with omega-6 fatty acids for five days; and 3, regular diet plus 100 mL/d of 10% omega-3 fatty acids for five days). The study concluded that omega-3 infusion is safe and effective in reducing SI in ACLF. Even 28-day LT-free survival was significantly higher in the group of patients who received omega-3 [71]. However, the evidence is limited as yet, and more studies, ideally multicenter randomized trials, are needed in order to evaluate whether a general recommendation for patients with ACLF can be provided.

A recent study by Moreau et al. was able to demonstrate that in ACLF patients SI is associated with blood metabolite accumulation as well as profound alterations in major metabolic pathways. The inhibition of mitochondrial energy production might especially contribute to the development of intra- and extrahepatic organ failure [72]. Approaches with liposome-supported peritoneal dialysis, especially for the extraction of ammonia and other potentially harmful metabolites, have been the topic of research in the past [73].

A poster presentation by Uschner et al. at AASLD in 2021 demonstrated the results of the phase-I-b clinical trial on VS-01, which is a novel intraperitoneal pH-gradient liposomal infusion drug that has been shown to enhance clearance of ammonia and other potentially harmful metabolites. It showed the safety and tolerability of the intraperitoneal application of VS-01. Furthermore, in this small group of patients it demonstrated promising clinical efficacy, with the results supporting future development of VS-01 for patients with ACLF and organ failures [74]. A trial on the application of VS-01 in ACLF in a larger cohort to evaluate its clinical efficacy is expected in the near future.

Furthermore, the intravenous application of human allogeneic liver-derived progenitor cells (HALPC) (HepaStem^®^; Promethera Biosciences, Mont-Saint-Guibert, Belgium) is currently being investigated for patients with AD and ACLF. Nevens et al. investigated the use of HALPC in 24 patients (nine AD, 15 ACLF), and were able to demonstrate a reduction of markers of SI and altered liver function in the surviving patients. The 28-day and 3-month survival rates were 83% and 71%, respectively, and no patient had ACLF at month three [75]. Currently, the DHELIVER study, an interventional double blind randomized and placebo-controlled phase-II-b study, is being performed to investigate the use of HALPC in patients recently diagnosed with ACLF grade 1 or 2 in a larger cohort (NCT04229901).

The results of these and further studies will show whether the newly developed substances named above can offer feasible treatment options for patients with ACLF.

## Data Availability

Not applicable.
